# Sulfates Increase the Antioxidant Activity of Cowpea Subjected to Saline Stress

**DOI:** 10.1021/acsomega.5c08126

**Published:** 2026-02-04

**Authors:** Vladimir Rubiano González, Alison Rocha de Aragão, Letycia de Lima Costa, Maria Aparecida dos Santos Morais, Lucilândia de Souza Bezerra, Jackson Silva Nóbrega, Nildo da Silva Dias, Patrícia Lígia Dantas de Morais

**Affiliations:** † Department of Agronomic and Forestry Sciences, 74384 Universidade Federal Rural Do Semi-Árido, Mossoró, Rio Grande Do Norte 59625-900, Brazil; ‡ Universidade Federal Do Oeste Do Pará, Rurópolis University Campus, Rurópolis, Pará 68165-000, Brazil

## Abstract

Water scarcity for irrigation presents significant challenges to agriculture. The use of saline water from deep wells can induce salt stress in crops, negatively impacting their growth and productivity. Nutritional management using sulfates has emerged as a promising strategy to mitigate the adverse effects of salinity. Therefore, this study aimed to evaluate the effect of sulfate application as a salt stress attenuator in cowpea plants (cv. BRS Tumucumaque). The experiment was conducted in a greenhouse using a randomized block design in a 2 × 4 factorial arrangement, consisting of two irrigation water electrical conductivity levels (ECw = 0.6 and 4.5 dS m^–1^) and four sulfate treatments: no sulfate (control), calcium sulfate, potassium sulfate, and ammonium sulfate. Irrigation with water at 4.5 dS m^–1^ reduced the cowpea growth and yield. However, it also stimulated pigment synthesis and the enzymatic activities of catalase (CAT) and ascorbate peroxidase (APX), particularly when combined with calcium and potassium sulfate, respectively. In contrast, ammonium sulfate intensified salt stress, increasing electrolyte leakage and lipid peroxidation in cowpea plants. Leaf Na^+^ concentrations remained stable, while calcium and potassium sulfate increased Mg^2+^ and K^+^ levels, respectively. The Na^+^/K^+^ ratio was lower with the potassium sulfate application. Overall, the application of calcium and potassium sulfates enhanced cowpea performance by alleviating the detrimental effects of salinity on growth, pigment production, yield, and antioxidant enzyme activity.

## Graphical abstract

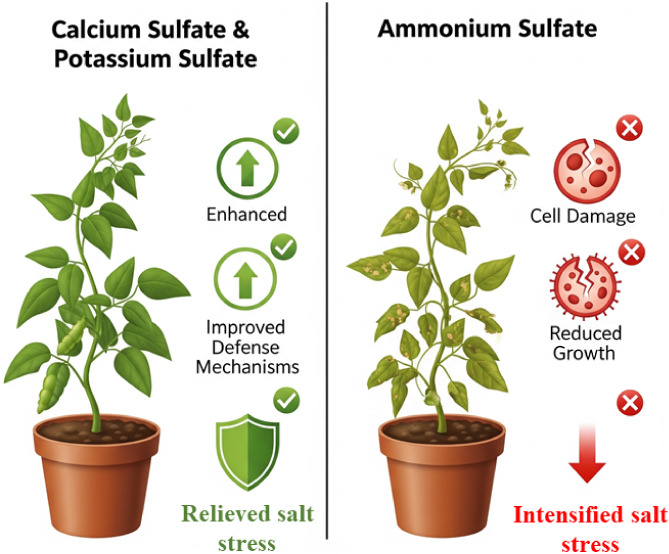

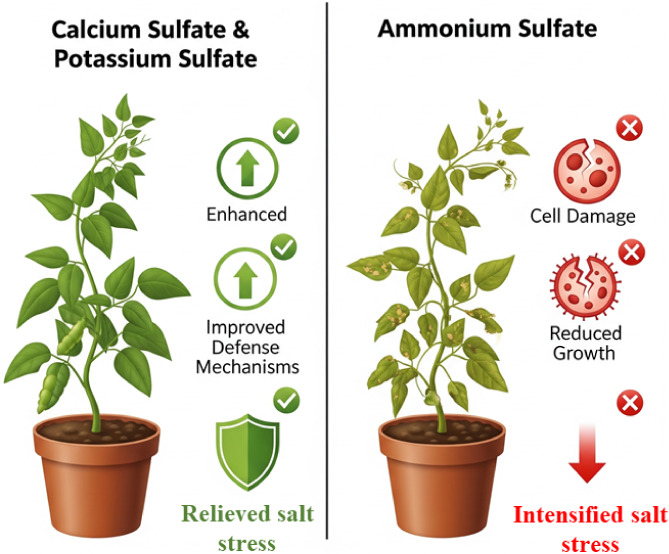

## 1 Introduction

Surface water availability for agricultural use is becoming increasingly limited due to the expansion of irrigated areas to meet global food demand and ensure water security. In semiarid regions, such as the Brazilian Northeast, groundwater is often used as an alternative to meet water needs. However, in most cases, these sources have usage limitations due to the risk of salinity-related issues.^1^

In plants, the primary negative effects of salt stress are both osmotic and ionic in nature. Osmotic stress occurs when the reduction in soil osmotic potential limits the plant’s ability to absorb water. Ionic stress, on the other hand, results from the toxicity of specific ions such as Na^+^ and Cl^–^, as well as from nutritional imbalances caused by the antagonistic effects of Na^+^, which inhibits the uptake of essential nutrients like K^+^, Ca^2+^, and Mg^2+^.^2–4^ In addition, salt stress triggers excessive production and accumulation of reactive oxygen species (ROS), leading to oxidative stress, which can cause denaturation of nucleic acids and proteins and impair enzyme synthesis and activity.^5^

Cowpea is classified as a salinity-tolerant species (3.3 dS m^–1^).^6^ Among the main cowpea cultivars, there is the BRS Tumucumaque cultivar, which has high adaptability to the environmental conditions of the Brazilian Northeast, with good performance observed in different studies under saline stress conditions. In a study carried out during the initial establishment phase, the cv. BRS Tumucumaque stood out as one of the most tolerant to saline stress.^7^ In other studies, the cultivar showed greater tolerance to salinity during the growth and production phases, proving to be a cultivar with a greater capacity to tolerate the deleterious effects of saline stress.^8,9^

However, depending on the salinity level of the irrigation water, yield losses may still occur. In such cases, it is essential to adopt additional management strategies to mitigate the effects of salt stress and ensure crop productivity in semiarid agricultural systems.^10^ The literature contains numerous reports indicating that the application of sulfur (S) in the form of sulfate (SO_4_^2–^), often combined with other minerals, such as calcium, potassium, magnesium, phosphorus, and ammonium, is widely used as a strategy to mitigate the effects of salt stress and nutrient deficiencies in plants. This approach enhances nutrient availability and uptake by plants.^11^

Furthermore, nutrient management involving sulfate can help alleviate the adverse effects of salinity by activating and/or enhancing plant defense mechanisms that confer salt stress tolerance.^12^ Among the effects that sulfur can promote in plants subjected to stress conditions, the biosynthesis of biochemical compounds and the activation of antioxidant enzymes, such as sulfite reductase, stand out. Sulfite reductase directly participates in the reductive pathway of sulfate assimilation, as well as in detoxification and protection against excessive sulfite accumulation.^13^ It also plays a role in the integration of metabolic compounds, such as amino acids like cysteine and methionine, enzymes, and proteins,^14^ in addition to interacting with phytohormones, helping in mineral balance, and acting as a signaling molecule in situations of abiotic stress.^15^ For example, the presence of sulfur-containing compounds is fundamental for the biosynthesis of abscisic acid (ABA), regulating stomatal opening when the plant is under stress.^16^

Thus, the hypothesis of this study is that the application of sulfate sources can enhance the production of osmoprotective compounds and boost the antioxidant activity of enzymes involved in the plant defense system, thereby alleviating the harmful effects of salt stress on cowpea plants cv. BRS Tumucumaque.

Thus, the objective of this study was to evaluate the mitigating effects of different sulfate sources on growth, photosynthetic pigment biosynthesis, biochemical activity, ionic balance, and yield of cowpea plants (cv BRS Tumucumaque) under salt stress.

## 2 Materials and Methods

### 2.1 Location of the Experimental Area

The experiment was carried out from March to May 2023 in a greenhouse with an area of 126 m^2^, 4 m high, covered with white plastic and 50% shade cloth walls, belonging to the Federal Rural University of Semiarid—UFERSA, in the municipality of Mossoró-RN (−5.200754 S, −37.3264682 W), at an altitude of 11 m. The greenhouse environment during the research is shown in Figure 1.

**Figure 1:**
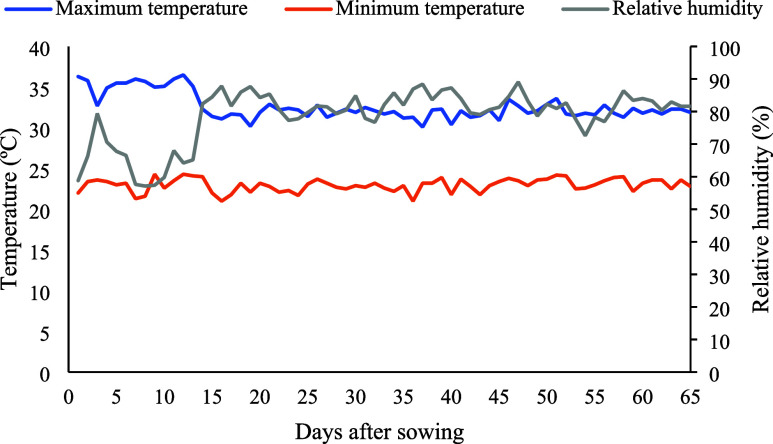
Temperature and relative humidity data from the greenhouse during the experiment.

### 2.2 Experimental Design

The experiment was conducted using a randomized complete block design in a 2 × 4 factorial arrangement. Treatments consisted of two levels of irrigation water salinity (EC_w_ = 0.6 and 4.5 dS m^–1^) and four sulfate sources for stress attenuation: no sulfate (S1), calcium sulfate (CaSO_4_·2H_2_O) (S2), potassium sulfate (K_2_SO_4_) (S3), and ammonium sulfate ((NH_4_)_2_ SO_4_) (S4), with seven replicates per treatment, totaling 56 experimental units. The sulfate sources were based on a study conducted with sunflower crops subjected to water deficit, based on their beneficial effects in mitigating the effects of stress.^16^

### 2.3 Conducting the Experiment

The experiment was conducted by using plastic bags with a capacity of 8 dm^3^, each filled with a layer of gravel at the bottom and a substrate composed of coconut fiber and soil in a 2:1 ratio. The soil was collected from the topsoil layer (0–30 cm) of a sandy-textured area at the UFERSA experimental farm. According to the classification,^17^ it was classified as a Dystrophic Red Latosol based on its chemical characteristics (Table 1).

**Table 1:** Chemical Characterization of the Topsoil Layer (0–30 cm) of a Dystrophic Red Latosol Used as a Substrate for Cowpea Cultivation^a^

pH (H_2_O)	ECse (dS m^–1^)	OM (g kg)	P (mg/dm^3^)	K^+^ (mg/dm^3^)	Na^+^ (mg/dm^3^)	Ca^2+^(cmolc/dm^3^)	Mg^2+^(cmolc/dm^3^)	Al^3+^ (cmolc/dm^3^)	H + Al (cmolc/dm^3^)	BS (cmolc/dm^3^)	t (cmolc/dm^3^)	CEC (cmolc/dm^3^)	V (%)	M (%)	PES (%)
7.4	0.80	31.9	112.2	791.7	160.1	6.5	4.8	0.0	0.0	14.0	14.5	14.0	100	0.0	5.5

a ECse = electrical conductivity of soil extract; OM = organic matter; (H + Al) = potential acidity; BS = sum of bases; t = effective CEC; CEC = soil CEC or CEC; V = base saturation; m = aluminum saturation; PES = percentage of exchangeable sodium.

The water with the lowest electrical conductivity (0.6 dS m^–1^) was used as is, while the saline water treatment (4.5 dS m^–1^) was obtained by mixing water from a deep well at UFERSA, which has an electrical conductivity of 5.2 dS m^–1^, with the 0.6 dS m^–1^ water supply to reach the target conductivity of 4.5 dS m^–1^. The chemical composition of the irrigation water is listed in Table 2.

**Table 2:** Chemical Characterization of Irrigation Water Used in the Experimental Cultivation^a^

	pH (H_2_O)	EC(dS m^–1^)	K^+^ (mmolc L)	Na^+^ (mmolc L)	Ca^2+^ (mmolc L)	Mg^2+^ (mmolc L)	Cl^–^ (mmolc L)	CO_3_^2–^ (mmolc L)	HCO_3_^–^ (mmolc L)	SAR
ABT	7.5	0.60	0.31	3.78	0.84	1.20	2.40	0.61	3.21	3.76
ASP	7.8	5.20	0.93	18.82	15.10	17.15	19.40	0.00	4.67	4.71

a ABT = supply water; ASP = saline well water; pH (H_2_O) = hydrogen potential in water; EC (dS m^–1^) = electrical conductivity; K^+^ = potassium; Na^+^ = sodium; Ca^2+^ = calcium; Mg^2+^ = magnesium; Cl^–^ = chloride; CO_3_^2–^ = carbonate; HCO_3_^–^ = bicarbonate; SAR = sodium adsorption ratio.

The experiment used seeds of cv. BRS Tumucumaque, with three seeds sown per bag. Thinning was carried out 10 days after sowing (DAS), when the plants had developed their first two pairs of true leaves, leaving one plant per bag. Fertilization management began simultaneously, using fertigation with a nutrient solution formulated according to the recommendations of Furlani^18^ (Table 3). The fertigation solutions were prepared and stored in 500 L plastic containers, which were covered to prevent contamination from rainwater and minimize evaporation.

**Table 3:** Nutrient Sources Used in the Experimental Solution^a^

Nutrient	mg L^–1^	Sources	Amount
N	147.35	Urea	0.112 g L^–1^
P	35	MAP	0.057 g L^–1^
K	189	KCl	0.315 g L^–1^
Ca	119	CaNO_3_	0.626 g L^–1^
Mg	28	MgSO_4_	0.311 g L^–1^
S	36.4	MgSO_4_	36.711 g L^–1^

Micronutrient	mg L^–1^	Sources	Amount
B	0.35	H_3_BO_3_	0.5 mL L^–1^
Cu	0.07	CuSO_4_	0.5 mL L^–1^
Mn	0.35	MnSO_4_	0.5 mL L^–1^
Mo	0.035	MoNO_4_	0.5 mL L^–1^
Zn	0.21	ZnSO4	0.5 mL L^–1^
Fe	1.54	FeSO_4_	0.5 mL L^–1^

a MAP = monoammonium phosphate; KCl = potassium chloride; CaNO_3_ = calcium nitrate; MgSO_4_ = magnesium sulfate; H_3_BO_3_ = boric acid; CuSO_4_ = copper sulfate; MnSO_4_ = manganese sulfate; MoNO_4_ = molybdenum mononitrate; ZnSO_4_ = zinc sulfate; FeSO_4_ = iron sulfate.

The different sulfate treatments were applied 14 days after sowing and 24 h before irrigation with saline water, at a concentration of 0.32 g per bag per day, totaling 20.8 g per plant. The sulfates were diluted in water and applied through daily fertigation, continuing until the cowpeas began flowering. When the plants reached the flowering stage (40 DAS), three plants from each treatment were collected for biometric analysis.

### 2.4 Variables Analyzed

At 42 DAS, the growth of BRS Tumucumaque cowpea plants was evaluated by measuring plant height (PH), stem diameter (SD), number of leaves (NL), leaf area (LA), and root length (RL). Plant height was measured from the plant collar to the apical bud using a tape measure graduated in centimeters (cm), with results expressed in cm. Stem diameter was measured 1 cm above the plant collar using a digital caliper with values expressed in millimeters (mm). The number of leaves (NL) corresponded to the count of the photosynthetically active leaves. Leaf area (LA) was determined using a LI-3100C Area Meter, with results expressed in cm^2^. Subsequently, the roots were removed from the substrate, washed, and gently dried with paper towels to remove excess water. Root length was measured with a ruler graduated in centimeters from the plant collar to the tip of the root system, with results expressed in cm.

At 42 DAS, the dry mass was determined by separating the plants into leaves, stem, and roots. These parts were packed in Kraft paper bags and dried in a forced-air oven at 65 °C until a constant weight was achieved. Afterward, the samples were weighed on an analytical balance with an accuracy of 0.0001 g to obtain the dry mass of the stem (DMS), leaves (DML), and roots (DMR). The dry mass partition among the different plant parts was calculated with values expressed in grams per plant.

At 42 DAS, photosynthetic pigment levels were determined using fully expanded leaves from four plants per treatment. Chlorophyll *a*, chlorophyll *b*, total chlorophyll, and carotenoid contents were measured. Leaves were collected, stored in plastic bags, placed in a Styrofoam box with ice, and transported to the Laboratory of Soil, Water, and Plant Analysis of the Semiarid Region (LASAPSA). Subsequently, leaf discs weighing 0.4 g were excised and placed in test tubes covered with aluminum foil to protect from light, containing 8 mL of 80% acetone, and kept in the dark for 5 h. After incubation, absorbance readings were taken using a spectrophotometer at wavelengths of 470 nm for carotenoids and 645, 652, and 663 nm for chlorophyll *a*, *b,* and total chlorophyll, respectively. Chlorophyll concentrations were determined following the methodology of Witham,^19^ while carotenoid content was calculated using the method of Lichtenthaler and Wellburn.^20^ Results were expressed as milligrams per gram of fresh leaf tissue (mg g^–1^ FW).

Electrolyte leakage was measured at 42 days after sowing using eight leaf discs collected from four plants per treatment. The discs were placed in test tubes containing 10 mL of deionized water and allowed to incubate for 24 h. The initial electrical conductivity (ECi) was then measured by using a benchtop conductivity meter. Subsequently, the tubes were heated in a water bath at 100 °C for one h, and the final electrical conductivity (ECf) was recorded. Electrolyte leakage was calculated following the methodology of Scotti-Campos.^21^

Biochemical activity was determined using leaf extracts from the third pair of leaves, located in the middle portion of the plant, which were frozen in liquid nitrogen. The enzymatic activities of catalase (CAT), peroxidase (POX), ascorbate peroxidase (APX), and superoxide dismutase (SOD), as well as the concentrations of hydrogen peroxide (H_2_O_2_), proteins, and malondialdehyde (MDA), were measured.

The extract was prepared by weighing 0.5 g of leaves and adding 25 mg of polyvinylpyrrolidone (PVPP), followed by maceration in liquid nitrogen. Subsequently, 1.5 mL of an acetate buffer solution (0.1 M, pH 5.0) containing 0.25 mL of 0.1 mM ethylenediaminetetraacetic acid (EDTA) was added. The mixture was homogenized, transferred to Eppendorf tubes, and centrifuged at 10,000 rpm for 10 min at 4 °C. The supernatant was then transferred to a fresh Eppendorf tube and stored in an ultralow temperature freezer (−80 °C) until analysis.

The concentration of total soluble proteins was determined using the method described by Bradford.^22^ In a cuvette, 20 μL aliquots of the extract were mixed with 1 mL of Bradford reagent, homogenized, and incubated in the dark for 10 min. Absorbance was measured at 595 nm. Protein concentration was calculated using a bovine serum albumin (BSA) standard curve.

Catalase (CAT) enzymatic activity was determined following the method of Havir and McHale,^23^ with modifications by Azevedo.^24^ In test tubes, 2.75 mL of potassium phosphate buffer (100 mM, pH 7.5), 100 μL of protein extract, and 120 μL of hydrogen peroxide (H_2_O_2_) solution were mixed. The reaction mixture was then transferred to quartz cuvettes, and the decrease in absorbance at 240 nm was measured using a spectrophotometer over a 60-s interval. CAT activity was calculated based on the rate of H_2_O_2_ decomposition and expressed as μmol min^–1^ mg^–1^ protein.

Peroxidase (POD) enzymatic activity was determined following the method described by Bezerra Neto and Barretos.^25^ In Eppendorf tubes, 25 μL of guaiacol (0.2 M), 250 μL of hydrogen peroxide (0.38 M), and 1 mL of sodium phosphate buffer (0.2 M, pH 6.0) were mixed and shaken. Then, 25 μL of the protein extract was added to quartz cuvettes. Absorbance readings were taken at 470 nm using a spectrophotometer over 1 min, with measurements every 10 s. Enzyme activity was calculated based on the change in absorbance per minute, normalized by sample weight, and expressed as EU min^–1^ of sample.

To determine the activities of ascorbate peroxidase (APX) and superoxide dismutase (SOD), protein extracts were prepared following the method of Azevedo.^24^ A sample of 370 mg of frozen leaf tissue was weighed along with 15% polyvinylpolypyrrolidone (PVPP) and macerated in liquid nitrogen. Then, 1100 μL of 100 mM potassium phosphate buffer (pH 7.5), supplemented with 1 mM ethylenediaminetetraacetic acid (EDTA) and 3 mM dithiothreitol (DTT), was added. The mixture was homogenized, transferred to Eppendorf tubes, and centrifuged at 10,000 rpm for 30 min at 4 °C.

To determine superoxide dismutase (SOD) activity, test tubes covered with aluminum foil were prepared with 2050 μL of 85 mM sodium phosphate buffer (pH 7.8), 250 μL of nitroblue tetrazolium chloride (NBT), 200 μL of EDTA, 250 μL of methionine, and 250 μL of riboflavin for the blank solution. For the sample tubes, 2000 μL of sodium phosphate buffer and 100 μL of protein extract were added, maintaining the same amounts of NBT, EDTA, methionine, and riboflavin. The sample tubes were exposed to intense light and incubated for 15 min at room temperature, while the blank tubes were kept in the dark. Absorbance was measured at 560 nm by using a spectrophotometer. SOD activity was calculated based on the enzyme’s ability to inhibit NBT photoreduction,^26^ and results are expressed as U min^–1^ mg protein^–1^.

Ascorbate peroxidase (APX) activity was measured following the method of Nakano and Asada.^27^ For the assay, 1300 μL of 80 mM potassium phosphate buffer (pH 7.0), 200 μL of 5 mM ascorbate, 200 μL of EDTA, and 100 μL of protein extract were mixed in test tubes and incubated in a water bath at 30 °C. At the time of measurement, 200 μL of hydrogen peroxide was added. Absorbance was recorded at 290 nm using a spectrophotometer with quartz cuvettes over a 1 min period. APX activity was quantified by monitoring ascorbate oxidation and expressed as μmol ascorbate min^–1^ mg protein^–1^.

Malondialdehyde (MDA) content was determined using the method of Heath and Packer,^28^ with some modifications. For this purpose, an extract was prepared by macerating 200 mg of fresh leaves in 0.1% trichloroacetic acid (TCA) combined with 20% polyvinylpolypyrrolidone (PVPP). The mixture was homogenized and centrifuged at 10,000 × g for 5 min at 4 °C. For the assay, 250 μL of the supernatant was mixed with 1.0 mL of a solution containing 0.5% thiobarbituric acid (TBA) and 20% TCA, incubated in a water bath at 95 °C for 30 min, and then cooled for 10 min. Absorbance readings were taken at 535 and 600 nm using a spectrophotometer with glass cuvettes.

Sodium (Na) and potassium (K) content in the leaves were analyzed by dry digestion, following the procedure described by Silva.^29^ Leaves were dried in an oven at 70 °C for 72 h and then ground using a Wiley stainless steel mill. A 0.25 g sample of the ground material was placed in Teflon tubes, to which 5 mL of nitric acid (HNO_3_) was added. The tubes were subjected to microwave digestion using the *Mars Xpress system* at 170 °C for 1 h. After the mixture cooled, 25 mL of deionized water was added, and the solution was filtered. Na^+^ and K^+^ concentrations were measured using a flame photometer, while Mg was determined by atomic absorption spectrometry. Results were expressed in grams kg^–1^ of dry matter (DM).

Cowpea production was evaluated at 65 DAS by measuring the number of pods per plant (NPP), pod mass per plant (PMP) in grams, pod length (PL) in centimeters, number of grains per pod (NGP), total number of grains per plant (TNG), total grain mass per plant (TGM) in grams, mass of 100 grains (M100G) in grams, and grain index (GI), with results expressed as percentages.

### 2.5 Statistical Analysis

Data were tested for normality using the Shapiro–Wilk test and subjected to analysis of variance (ANOVA) using the F test (*p* ≤ 0.05). When significant differences were detected, treatment means were compared using Tukey’s test (*p* ≤ 0.05). All analyses were performed using the Sisvar statistical software, version 5.6.^30^

## 3 Results

The height of cowpea plants was reduced by a salinity of 4.5 dS m^–1^, regardless of treatment, with the highest values (107.4 and 99.28 cm) in plants subjected to ammonium sulfate application and the control (Figure 2A), with reductions of 47.14 and 45.77%, respectively, when compared to the values of the lowest salinity (0.6 dS m^–1^). For calcium and potassium sulfate, there were decreases of 28.53 and 29.43%, respectively, when subjected to an ECw of 4.5 dS m^–1^.

**Figure 2:**
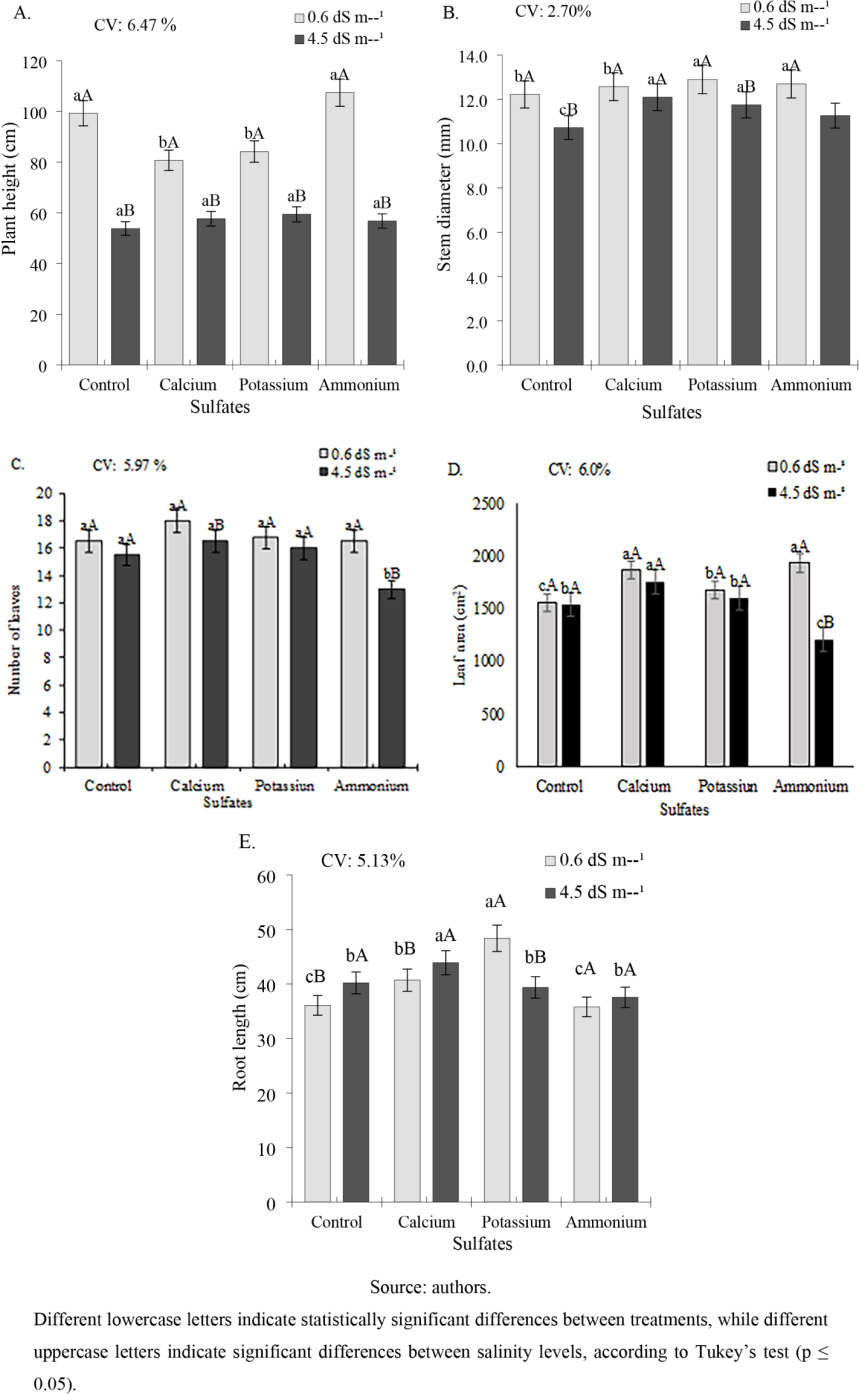
Plant height (A), stem diameter (B), number of leaves (C), leaf area (D), and root length (E) of cowpea cv. BRS Tumucumaque subjected to irrigation with water of different salinity levels and sulfate applications, 42 days after sowing.

For the stem diameter (Figure 2B), the application of different sulfate sources resulted in statistically higher values than the control, at both salinity levels. At an ECw of 0.6 dS m^–1^, stem diameters of 12.57, 12.90, and 12.70 mm were observed for calcium, potassium, and ammonium sulfate treatments, respectively. At an ECw of 4.5 dS m^–1^, the values were 12.09, 11.75, and 11.26 mm for the same sulfate sources. In contrast, the control plants showed stem diameters of 12.22 and 10.72 mm at ECw levels of 0.6 and 4.5 dS m^–1^, respectively.

The number of leaves did not differ significantly between treatments when plants were irrigated with water at an ECw of 0.6 dS m^–1^, with the highest value observed in the calcium sulfate treatment (18 leaves) (Figure 2C). Under irrigation with water at an ECw of 4.5 dS m^–1^, the control, calcium sulfate, and potassium sulfate treatments showed significantly higher leaf numbers compared to those of ammonium sulfate, with values of 15.5, 16.5, 16.0, and 13.0 leaves, respectively.

In the leaf area (Figure 2D), it is possible to verify that the plants subjected to ECw of 0.6 dS m^–1^ had superior values (1868.95 and 1975.33 cm^2^) when they received calcium and ammonium sulfate. Potassium sulfate (1679.74 cm^2^) was also significantly higher than that of the control (1557.39 cm^2^). In plants irrigated with water at an ECw of 4.5 dS m^–1^, calcium sulfate showed a significantly greater leaf area (1758.46 cm^2^) than the other treatments, representing increases of 14.23%, 10.03%, and 45.52% compared to the control, potassium sulfate, and ammonium sulfate treatments, respectively.

For root length (Figure 2E), at an ECw of 0.6 dS m^–1^, potassium sulfate resulted in the greatest root length (48.37 cm), followed by calcium sulfate (40.70 cm), representing increases of 34.1% and 12.84%, respectively, compared with the control treatment. Under an ECw of 4.5 dS m^–1^, calcium sulfate (4.90 cm) was statistically superior to the other treatments, with gains of 9.20%, 10.44%, and 16.91% relative to the control, potassium sulfate, and ammonium sulfate treatments, respectively.

For the relative growth rate of plant height (RGRPH), at an ECw of 0.6 dS m^–1^, all sulfate sources showed higher values than the control, with rates of 0.085, 0.094, and 0.098 cm cm^–1^ day^–1^ for calcium, potassium, and ammonium sulfate, respectively (Figure 3A). These represent increases of 63.5%, 80.77%, and 88.46% compared to the control. However, at an ECw of 4.5 dS m^–1^, calcium and potassium sulfate treatments did not differ statistically from the control and were superior only to ammonium sulfate.

**Figure 3:**
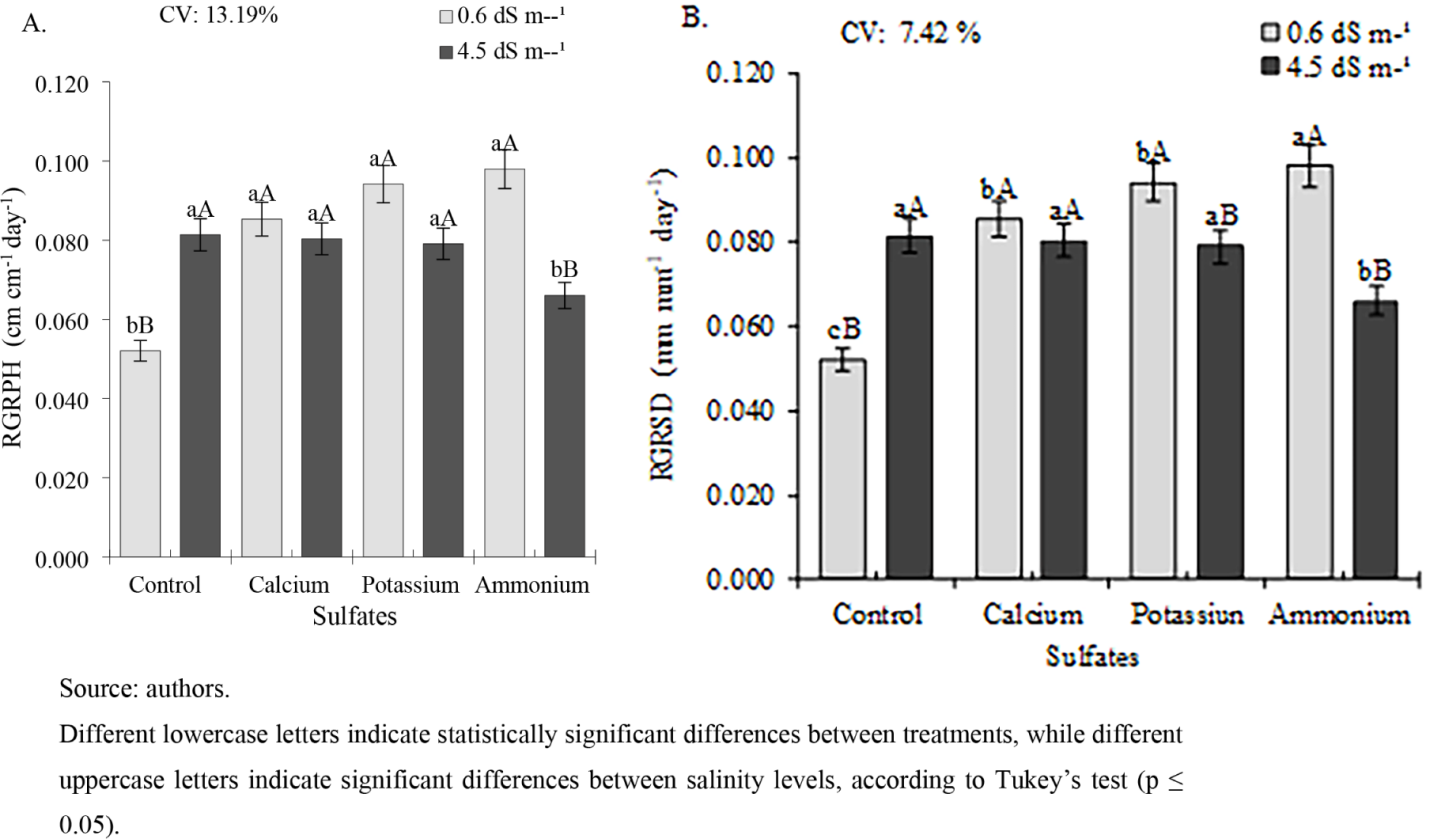
Relative growth rate of plant height (RGRPH) (A) and stem diameter (RGRSD) (B) of cowpea cv. BRS Tumucumaque subjected to irrigation with water of different salinity levels and sulfate applications, 35–42 days after sowing.

The relative growth rate of stem diameter (RGRSD) in cowpea plants showed a pattern similar to that of RGRPH. At an ECw of 0.6 dS m^–1^, all sulfate treatments were superior to the control, with ammonium sulfate exhibiting the highest value (0.097 mm mm^–1^ day^–1^), significantly outperforming all other treatments (Figure 3B). Calcium and potassium sulfates were also statistically higher (0.094 and 0.085 mm mm^–1^ day^–1^, respectively) than that of the control (0.052 mm mm^–1^ day^–1^). Under an ECw of 4.5 dS m^–1^, the control, calcium sulfate, and potassium sulfate treatments did not differ significantly from each other but were all superior to that of ammonium sulfate.

For leaf dry mass (LDM), no significant differences were observed between treatments in plants irrigated with water at an ECw of 0.6 dS m^–1^, although the highest value (12.44 g per plant) was recorded in plants treated with calcium sulfate (Figure 4A). Under irrigation with water at an ECw of 4.5 dS m^–1^, calcium and potassium sulfate treatments resulted in significantly higher LDM (13.77 and 12.59 g per plant, respectively), representing increases of 19.11% and 8.91% compared to the control (11.56 g per plant).

**Figure 4:**
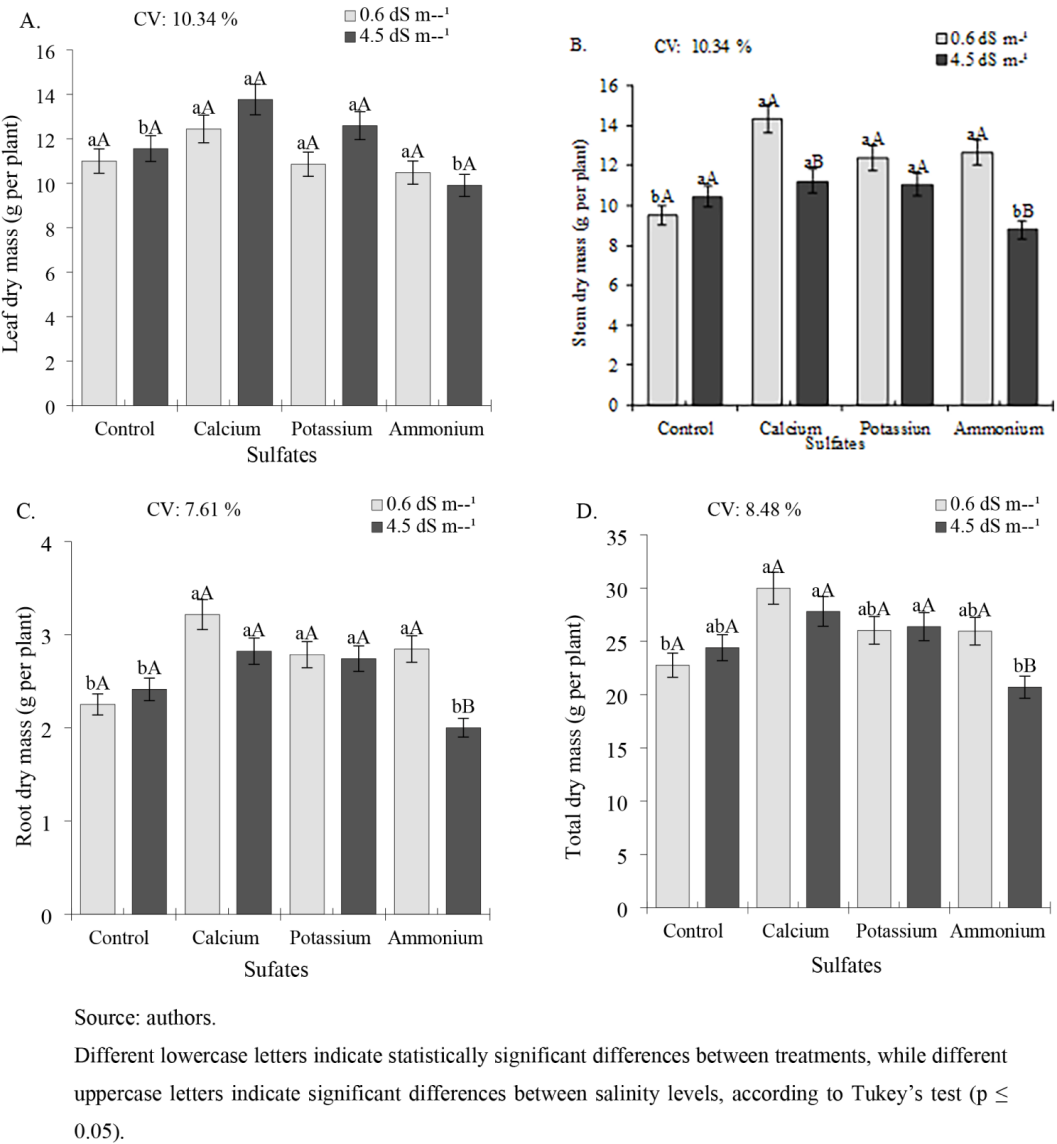
Dry mass of leaves (LDM) (A), stem (SDM) (B), root (RDM) (C), and total dry mass (TDM) (D) of cowpea cv. BRS Tumucumaque subjected to irrigation with water of different salinity levels and sulfate applications, 42 days after sowing.

Stem dry mass (SDM) was significantly higher in plants subjected to an ECw of 0.6 dS m^–1^, with increases of 50.52%, 30.14%, and 32.67% observed following the application of calcium, potassium, and ammonium sulfates, respectively, compared to the control treatment (Figure 4B). Under the higher salinity level of 4.5 dS m^–1^, there were no significant differences among the control, calcium sulfate, and potassium sulfate treatments (10.43, 11.23, and 11.04 g per plant, respectively), although all were superior to ammonium sulfate (8.78 g per plant).

For root dry mass (RDM), when plants were subjected to salinity of 0.6 dS m^–1^, the treatments with calcium, potassium, and ammonium sulfates (3.21, 2.78, and 2.84 g per plant) were superior, with observed increases of 43.11, 24.0, and 26.66%, respectively, compared to the control (2.25 g per plant) (Figure 4C). Under higher salinity conditions (4.5 dS m^–1^), plants treated with calcium and potassium sulfates (2.82 and 2.74 g per plant, respectively) were statistically superior to the other treatments, promoting increases of 17.01% and 13.69%, respectively, compared to the control (2.41 g per plant).

The total dry mass (TDM) of bean plants subjected to ECw of 0.6 dS m^–1^ was higher in the treatments that received the application of calcium, potassium, and ammonium sulfate (29.99, 26.03, and 25.95 g per plant), providing gains of 31.76, 14.36, and 14.04%, respectively, in relation to the control (22.76 g per plant) (Figure 4D). Under the highest salinity level (4.5 dS m^–1^), there was no statistical difference between the control, calcium sulfate, and potassium sulfate treatments (24.40, 27.80, and 26.37 g per plant), although all were superior to ammonium sulfate (20.69 g per plant).

For chlorophyll *a* content (Figure 5A), in plants irrigated with a ECw of 0.6 dS m^–1^, potassium and ammonium sulfate treatments showed statistically higher values (17.61 and 19.40 mg g^–1^ FW, respectively) compared to the others, representing increases of 17.25% and 29.25% relative to the control. Under the highest salinity level (4.5 dS m^–1^), the control, calcium sulfate, and ammonium sulfate (18.34, 19.13, and 17.77 mg g^–1^ FW, respectively) treatments did not differ significantly from one another but were superior to potassium sulfate (16.70 mg g^–1^ FW).

**Figure 5:**
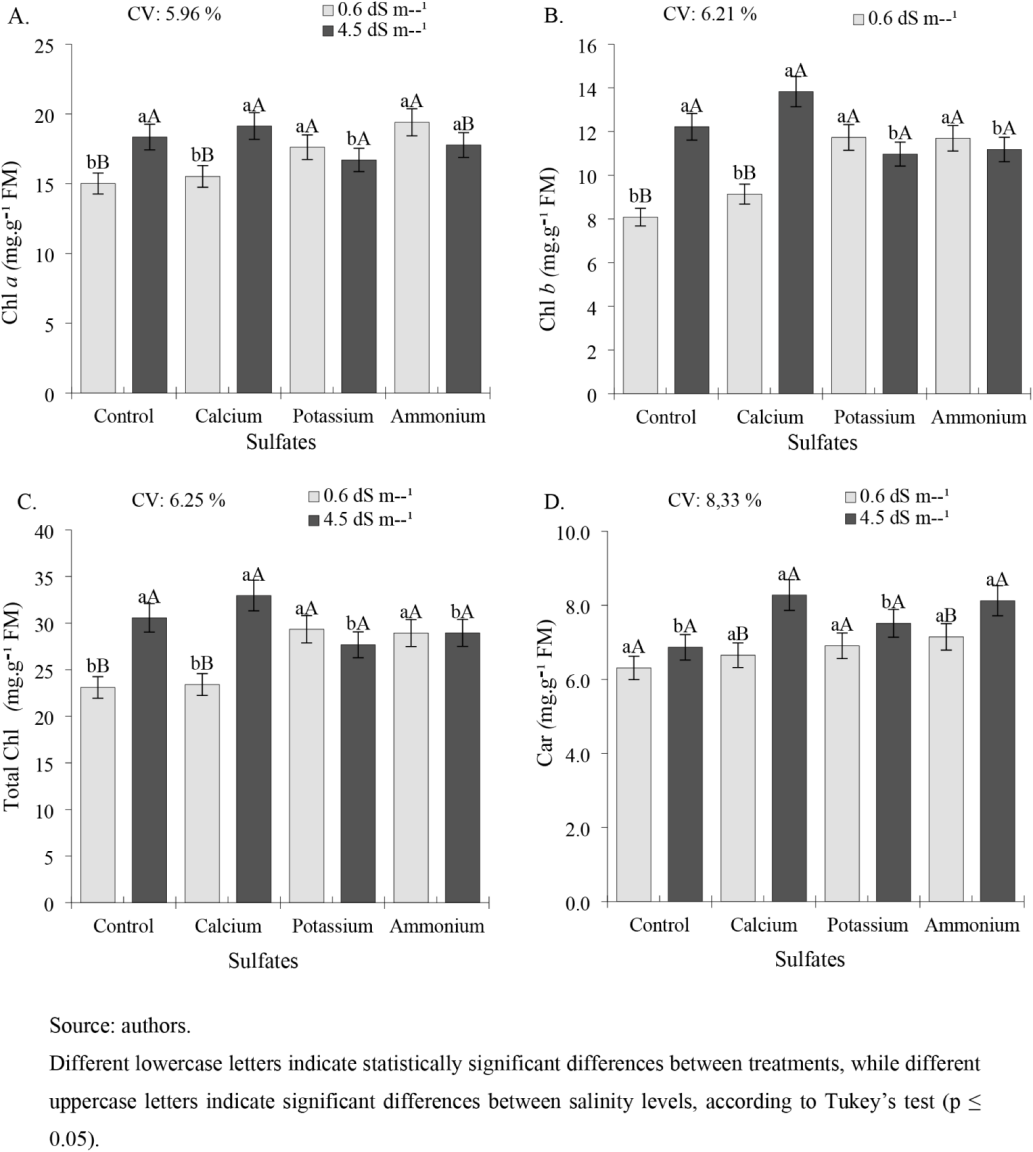
Chlorophyll *a*—Chl *a* (A), chlorophyll *b*—Chl *b* (B), total chlorophyll—Total Chl (C), and carotenoids—Car (D) in cowpea cv. BRS Tumucumaque irrigated with water of different salinity levels and treated with sulfate sources, 42 days after sowing.

Chlorophyll *b* content (Figure 5B) in plants subjected to an ECw of 0.6 dS m^–1^ was higher with the application of potassium and ammonium sulfates (11.73 and 11.69 mg g^–1^ FW, respectively), representing increases of 45.17% and 44.67% compared to the control (8.08 mg g^–1^ FW). Under an ECw of 4.5 dS m^–1^, the highest Chl *b* value was observed in plants treated with calcium sulfate (13.83 mg g^–1^ FW), although it did not differ statistically from the control (12.22 mg g^–1^ FW); both were superior to the potassium and ammonium sulfate treatments (10.96 and 11.17 mg g^–1^ FW).

Total chlorophyll content (Figure 5C) was higher in plants subjected to an ECw of 4.5 dS m^–1^ under calcium sulfate treatment (32.96 mg g^–1^ FW) and in the control (30.56 mg g^–1^ FW), both statistically superior to the other treatments. Under low salinity conditions (0.6 dS m^–1^), the potassium and ammonium sulfate treatments showed statistically higher values (29.35 and 28.93 mg g^–1^ FW, respectively), resulting in increases of 27.11% and 25.29% compared to the control (23.09 mg g^–1^ FW).

For the carotenoid content (Figure 5D), there were no significant differences among treatments under an ECw of 0.6 dS m^–1^. However, under a salinity level of 4.5 dS m^–1^, calcium and ammonium sulfate treatments showed statistically higher values (8.28 and 8.12 mg g^–1^ FW, respectively), resulting in increases of 20.52% and 18.2% compared to the control (6.30 mg g^–1^ FW).

Superoxide dismutase (SOD) activity, under low salinity conditions (0.6 dS m^–1^), was higher when calcium sulfate (15.92 UE min^–1^ mg of protein^–1^) was applied, surpassing the other treatments, followed by potassium sulfate and the control (13.80 and 11.76 UE min^–1^ mg of protein^–1^), with the lowest SOD activity in plants subjected to ammonium sulfate (Figure 6A). Under a salinity of 4.5 dS m^–1^, the highest SOD activity occurred in plants that received calcium sulfate (12.33 UE min^–1^ mg of protein^–1^), followed by the control (11.20 UE min^–1^ mg of protein^–1^), both statistically superior to the other treatments.

**Figure 6:**
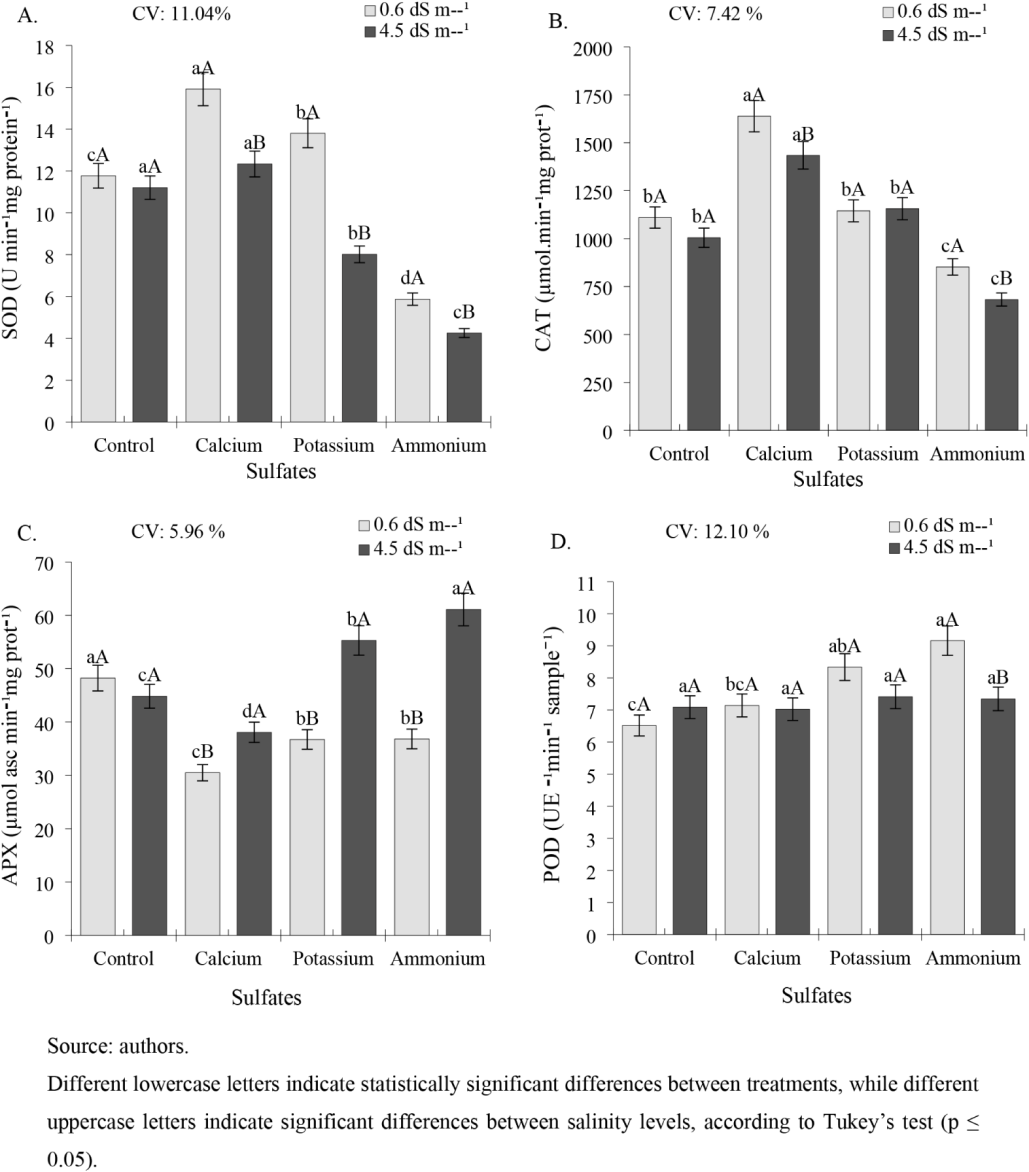
Activities of superoxide dismutase (SOD) (A), catalase (CAT) (B), ascorbate peroxidase (APX) (C), and peroxidase (POD) (D) in cowpea plants cv. BRS Tumucumaque subjected to irrigation with water of different salinities and sulfate applications, 42 days after sowing.

For catalase (CAT), calcium sulfate stimulated enzyme activity at both ECw levels of 0.6 and 4.5 dS m^–1^, showing the highest values (1,638.75 and 1,434.41 μmol min^–1^ mg^–1^ protein), representing increases of 47.64% and 42.78% compared to the control (1,109.97 and 1,004.61 μmol min^–1^ mg^–1^ protein), at ECw levels of 0.6 and 4.5 dS m^–1^, respectively (Figure 6B). Similar to the results observed for SOD, the lowest CAT activity was recorded in plants treated with ammonium sulfate, especially under an ECw of 4.5 dS m^–1^ (682.37 μmol min^–1^ mg^–1^ of protein).

Regarding ascorbate peroxidase (APX) activity, an increase was observed at a salinity level of 4.5 dS m^–1^ with sulfate treatments (Figure 6C), showing the highest APX values (61.09 and 55.29 μmol asc min^–1^ mg protein^–1^) in plants treated with ammonium sulfate and potassium sulfate, respectively. Under an ECw of 0.6 dS m^–1^, the control treatment showed the highest APX activity (61.09 μmol asc min^–1^ mg protein^–1^), statistically surpassing the others.

For the peroxidase enzyme (POD), the highest activity was observed in plants under an ECw of 0.6 dS m^–1^ when treated with ammonium and potassium sulfate (9.16 and 8.33 UE min^–1^ sample^–1^), differing significantly from the other treatments (Figure 6D). At a salinity of 4.5 dS m^–1^, no statistical differences were observed between the treatments.

Electrolyte leakage was higher in plants subjected to a salinity of 4.5 dS m^–1^, regardless of treatment (Figure 7A), with the highest values observed in the control (92.42%) and ammonium sulfate treatment (97.53%). Under low salinity conditions (0.6 dS m^–1^), plants treated with ammonium sulfate and potassium sulfate showed the greatest electrolyte leakage (80.71% and 76.38%, respectively).

**Figure 7:**
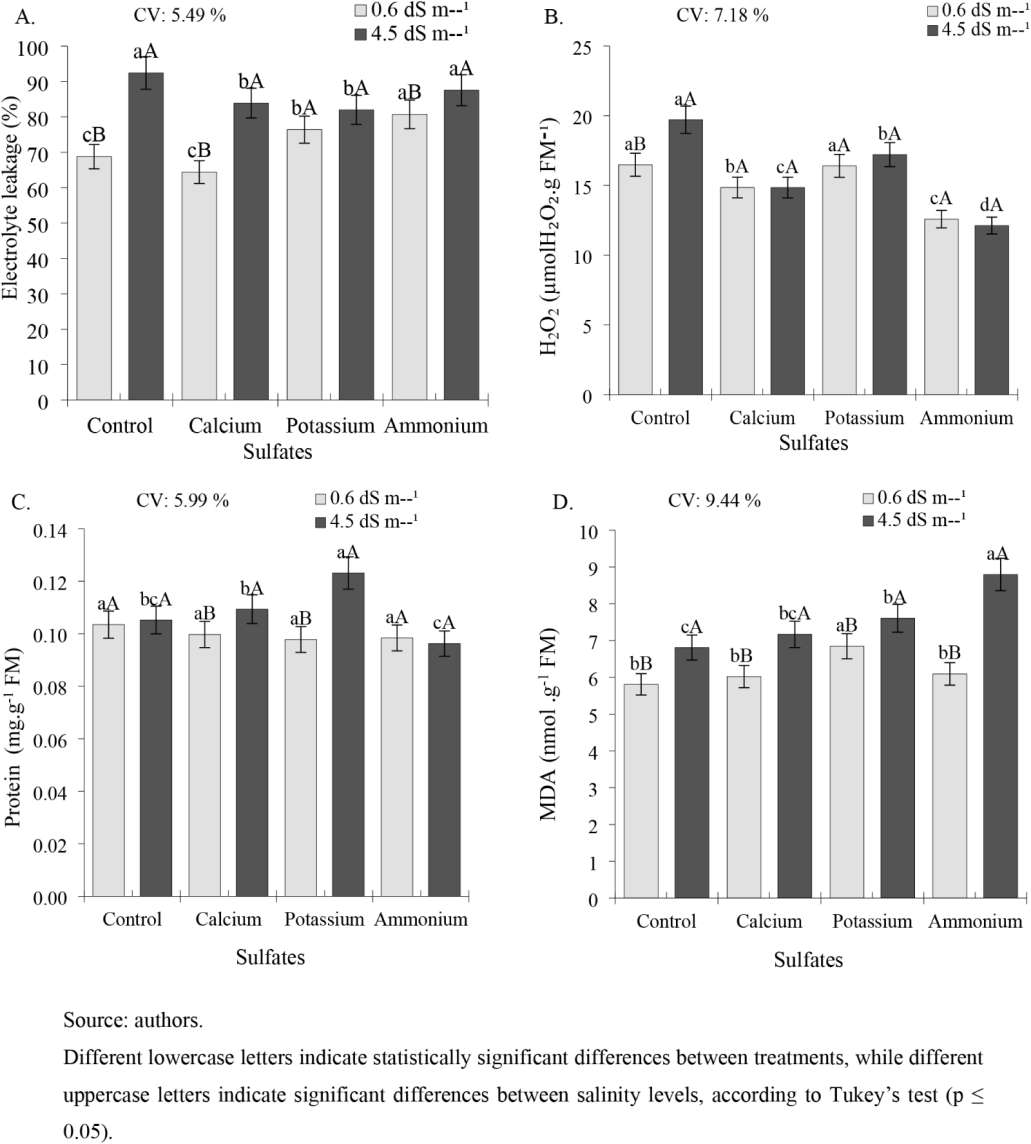
Electrolyte leakage (EE%) (A), hydrogen peroxide accumulation (H_2_O_2_) (B), protein content (C), and lipid peroxidation (MDA) (D) in leaves of cowpea cv. BRS Tumucumaque subjected to irrigation with water of different salinity levels and sulfate applications, 42 days after sowing.

The accumulation of H_2_O_2_ in bean leaves was highest in the control plants, both at an ECw of 0.6 dS m^–1^ (16.48 μmol H_2_O_2_ g^–1^ FW), not significantly different from the potassium sulfate treatment (16.40 μmol H_2_O_2_ g^–1^ FW), and at 4.5 dS m^–1^ (19.71 μmol H_2_O_2_ g MF^–1^), where it was statistically higher than the other treatments (Figure 7B).

Regarding protein content (Figure 7C), the highest value (0.12 mg g^–1^ FW) was found in plants subjected to an ECw of 4.5 dS m^–1^ and treated with potassium sulfate, statistically surpassing the other treatments. Under a salinity of 0.6 dS m^–1^, no significant differences were observed between treatments.

Lipid peroxidation (MDA) was stimulated by a salinity of 4.5 dS m^–1^ and sulfate applications, with the highest values (8.79, 7.60, and 7.17 nmol g^–1^ FW) observed in plants treated with ammonium, potassium, and calcium sulfate, respectively (Figure 7D). Under an ECw of 0.6 dS m^–1^, potassium sulfate treatment showed a higher value (6.85 nmol g^–1^ FW) compared to the other treatments.

Regarding ionic relations in bean leaves, no significant differences were observed in sodium (Na^+^) accumulation between treatments, with the highest values of 1.54 g kg^–1^ DM found in the control and in the calcium, potassium, and ammonium sulfate treatments (Figure 8A).

**Figure 8:**
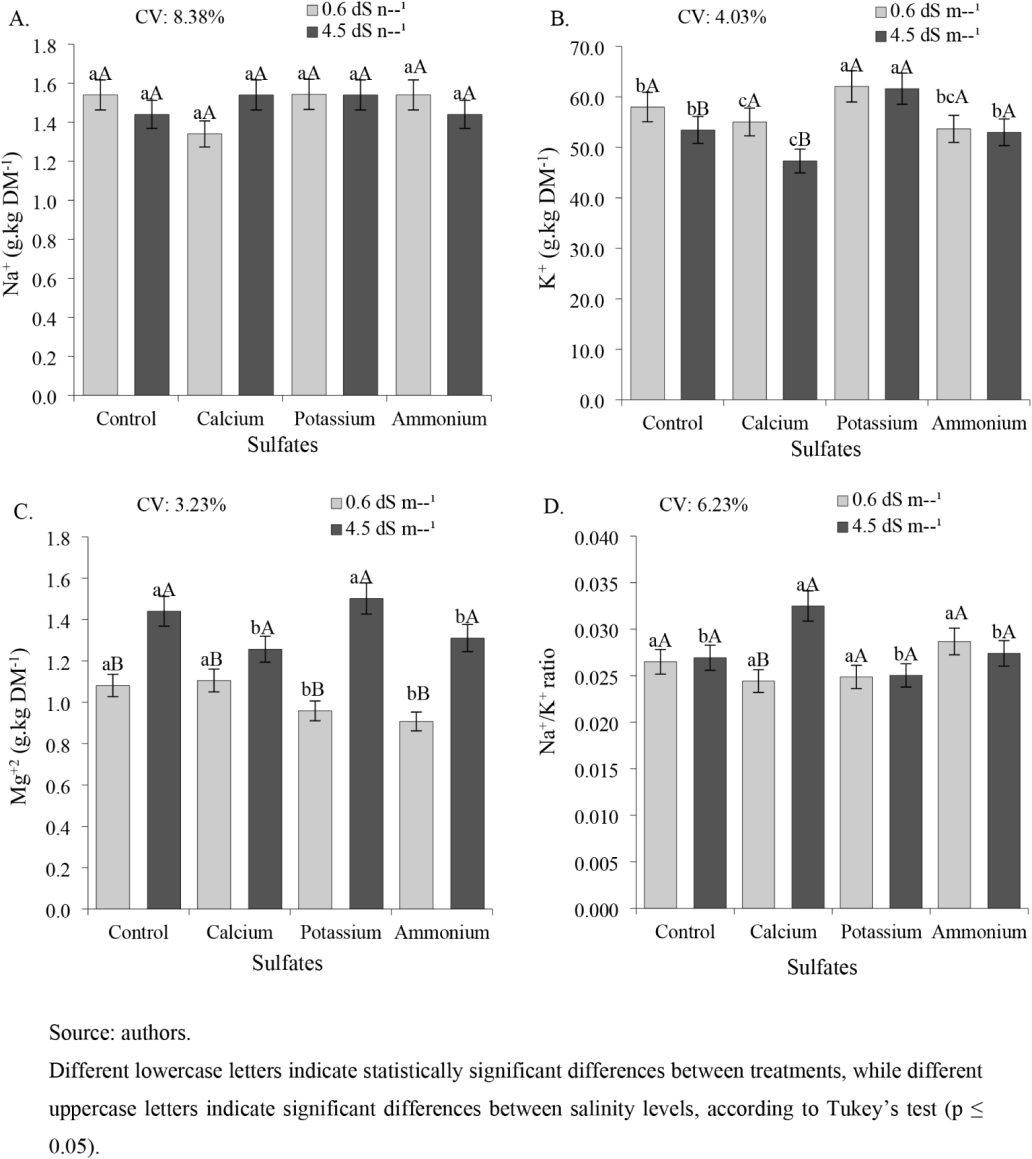
Accumulation of sodium (Na^+^) (A), potassium (K^+^) (B), magnesium (Mg^2+^) (C), and Na^+^/K^+^ ratio (D) in the leaves of cowpea cv. BRS Tumucumaque subjected to irrigation with water of different salinity levels and sulfate applications, 42 days after sowing.

For foliar potassium (K^+^) levels, plants treated with potassium sulfate showed the highest values, with 62.09 and 61.63 g kg^–1^ DM at ECw values of 0.6 and 4.5 dS m^–1^, respectively (Figure 8B). It was also observed that K^+^ levels decreased following calcium sulfate application, especially at an ECw of 4.5 dS m^–1^.

The magnesium content (Mg^2+^) was consistently higher in plants exposed to elevated ECw (4.5 dS m^–1^), with peak values observed in both the potassium sulfate treatment (1.50 g kg^–1^ DM) and the control (1.44 g kg^–1^ DM) (Figure 8C). In contrast, under the lower ECw (0.6 dS m^–1^), calcium sulfate and the control treatments showed significantly greater Mg^2+^ accumulation (1.11 and 1.08 g kg^–1^DM, respectively) compared to other treatments.

The Na^+^/K^+^ ratio (Figure 8D) showed significant differences only in plants exposed to higher salinity (ECw = 4.5 dS m^–1^). In this group, calcium sulfate treatment resulted in a significantly greater ratio (0.032) compared to all other treatments. No significant differences were observed among treatments at a lower salinity level (0.6 dS m^–1^).

The production components of cowpea were significantly reduced under high salinity (4.5 dS m^–1^) across all of the evaluated parameters. For pod number per plant (NPP), plants exposed to 4.5 dS m^–1^ ECw and treated with calcium sulfate or potassium sulfate showed the highest values (12 and 10 pods per plant, respectively), representing significant increases of 44.05% and 20% compared to the control (Figure 9A). These results were statistically different from those of other treatments. Under low salinity conditions (0.6 dS m^–1^), no significant differences were observed among treatments, though the numerically highest values (13.33 and 13 pods per plant) occurred with ammonium sulfate and potassium sulfate, respectively.

**Figure 9:**
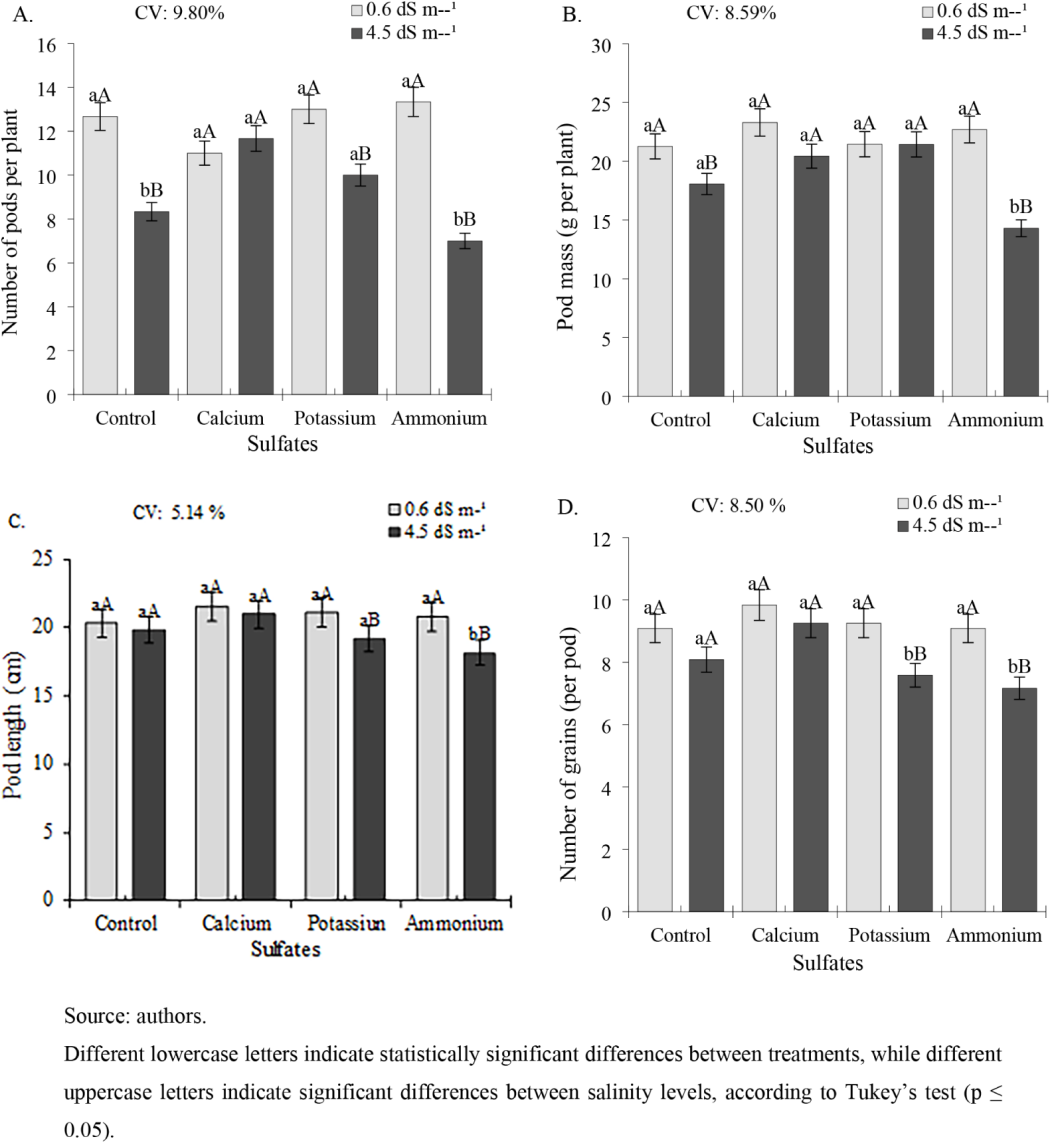
Number of pods per plant, NPP (A); pod mass per plant, PMP (B); pod length, PL (C); and number of grains per pod, NGP (D) of cowpea cv. BRS Tumucumaque subjected to irrigation with water of different salinities and application of sulfates, 42 days after sowing.

For pod mass per plant (PMP), significant differences were observed only under high salinity conditions (4.5 dS m^–1^ ECw). At this salinity level, the control, calcium sulfate, and potassium sulfate treatments showed significantly higher values (18.07, 20.43, and 21.43 g plant^–1^, respectively) compared to those of ammonium sulfate treatment (14.30 g plant^–1^). No significant differences were detected among treatments at the lower salinity level (0.6 dS m^–1^) (Figure 9B).

Pod length followed a similar pattern to that of PMP, showing significant treatment differences only at high salinity (4.5 dS m^–1^). Under these conditions, the control (20.32 cm), calcium sulfate (21.52 cm), and potassium sulfate (21.09 cm) treatments significantly outperformed the treatment with ammonium sulfate (Figure 9C). No treatment effects were observed at the lower salinity level (0.6 dS m^–1^).

The number of grains was generally higher in plants grown at 0.6 dS m^–1^ ECw, though no significant differences were observed among treatments at this salinity level (Figure 9D). Under high salinity conditions (4.5 dS m^–1^), calcium sulfate treatment yielded significantly more grains (9.25 per pod) than other treatments, followed by the control (8.08 pod^–1^), with both showing statistical superiority over the remaining treatments.

The number of grains per 5 pods was significantly reduced by high salinity (4.5 dS m^–1^ ECw) in the control, calcium sulfate, and ammonium sulfate treatments (Figure 10A). Potassium sulfate maintained consistent performance across both salinity levels, achieving the highest yield (68 grains plant^–1^) at 4.5 dS m^–1^—an 18.61% increase over the control. No treatment differences were observed at the lower salinity level (0.6 dS m^–1^).

**Figure 10:**
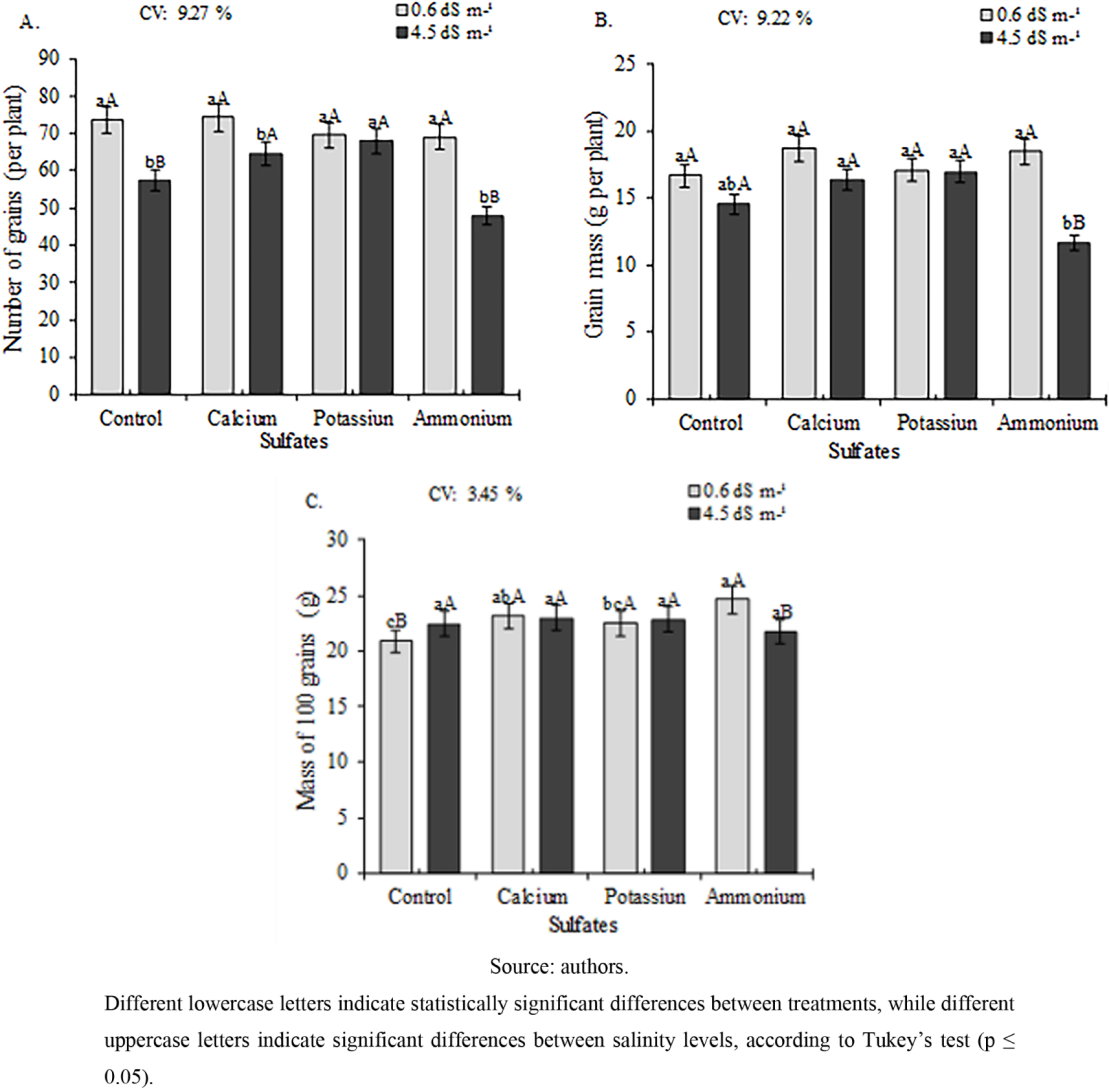
Number of grains per plant, NGP (A), grain mass per plant, MGP (B), and mass of 100 grains, M100G (C) of cowpea cv. BRS Tumucumaque subjected to irrigation with water of different salinities and application of sulfates, 65 days after sowing.

For grain mass per plant (MGP), significant differences among treatments were observed only at the higher salinity level (4.5 dS m^–1^ ECw). Calcium sulfate and potassium sulfate treatments yielded the highest values (16.37 and 16.99 g of plant^–1^, respectively), which were statistically superior to all other treatments (Figure 10B).

The 100-grain weight (Figure 10C) was significantly higher in plants grown at 0.6 dS m^–1^ ECw, with potassium sulfate (24.64 g) and calcium sulfate (23.17 g) treatments yielding the greatest values, which were statistically superior to those of other treatments. No significant treatment effects were observed at a higher salinity level (4.5 dS m^–1^).

## 4 Discussion

The increased salinity of irrigation water negatively affected cowpea growth, biomass accumulation, and yield, primarily due to the harmful effects of excess salts. Under saline stress, plants experience osmotic stress that limits their ability to absorb water and nutrients,^31^ causing loss of cell turgor and impairing various physiological processes, such as pigment biosynthesis and photosynthetic efficiency, resulting in growth losses, as observed in *Phaseolus vulgaris* L. plants.^32^

The second phase of the adverse effects of saline stress is of ionic origin, caused by specific ions that, in high concentrations, become toxic and interfere with the uptake of other essential nutrients. In particular, excess Na^+^ competes with K^+^ and Ca^2+^ for binding sites in the soil, leading to nutritional imbalances in the plant.^33^ Additionally, salinity stress promotes the excessive generation and accumulation of reactive oxygen species, triggering oxidative stress that damages nucleic acids, denatures proteins, and impairs enzymatic activity across various physiological processes.^34^

The adverse effects of salt stress significantly impaired the growth of cowpea plants. One of the most affected processes is cell division and expansion, leading to a reduction in plant height and stem diameter. In addition, salt stress disrupts key physiological functions—such as by reducing stomatal conductance—which limits carbon dioxide uptake and subsequently decreases photosynthetic activity.^35–37^ As a result, the production of photoassimilates is reduced, ultimately reducing phytomass accumulation in cowpea plants.

On the other hand, the application of calcium sulfate showed a mitigating effect on cowpea plants exposed to higher salinity levels (4.5 dS m^–1^), resulting in increased stem diameter, leaf area, root length, and dry mass of both leaves and roots. This indicates the beneficial effect of calcium sulfate fertilization under saline conditions. Calcium plays a crucial role in cell growth, particularly in the apical meristems of shoots and roots.^38^ Therefore, calcium sulfate application may have enhanced cellular activity, leading to greater growth responses compared with other treatments.

Total chlorophyll biosynthesis was stimulated by increased salinity, which may reflect an adaptive mechanism in cowpea plants to cope with salt stress. This response could be associated with an increase in chloroplast volume, aimed at enhancing light absorption and managing reactive oxygen species (ROS) production.^39^ In addition, higher levels of chlorophyll *b* and carotenoids were observed, particularly in treatments with calcium sulfate. These pigments serve as accessory components of the photosynthetic apparatus, supporting its function. Under stress conditions, they also contribute to the plant’s antioxidant defense system by scavenging ROS generated by salt stress.^40^

During sulfur assimilation, there is high activity of the enzymes ATP sulfurylase and ATP reductase, which act in the transformation of sulfate until its incorporation in the form of sulfide into amino acids such as cysteine, a precursor to the synthesis of glutathione, a compound that improves the physiological performance of plants, increasing the activity of photosynthetic pigments, the intrinsic efficiency of water use, and photosynthetic activity, providing improvements in plant growth under stress conditions.^41^

Enzymatic activity serves as a reliable indicator of the effects of salt stress, particularly for catalase (CAT) and ascorbate peroxidase (APX), which showed heightened responsiveness at an electrical conductivity of irrigation water (ECw) of 4.5 dS m^–1^. This response was further enhanced by the application of calcium sulfate, which resulted in the highest levels of enzyme expression. The increased activity of these enzymes can be interpreted as a plant defense mechanism aimed at enhancing the antioxidant system, thereby mitigating the effects of salt stress.^42,43^ The role of sulfates in stimulating enzyme activity may be attributed to the presence of sulfur, which is a key component of amino acids and proteins involved in phytohormone signaling pathways. These signaling molecules regulate various physiological processes and play a crucial role in mediating plant responses to environmental stressors.^15^

Elevated ROS production also affects enzyme activity and damages plant tissues, as evidenced by increased electrolyte leakage and lipid peroxidation in common bean plants. Excessive ROS leads to greater membrane permeability, causing structural and functional damage to cellular membranes and resulting in oxidative stress. To counteract this, plants must enhance antioxidant enzyme activity to scavenge and neutralize the excess ROS.^44^

Conversely, the application of sulfates—particularly calcium and potassium—was effective in reducing membrane damage, as indicated by lower electrolyte leakage and reduced H_2_O_2_ content. This suggests a beneficial effect of sulfate fertilization in mitigating the effects of salt stress. Calcium (Ca^2+^) is essential for maintaining membrane and cell wall integrity, and it also plays a signaling role in activating genes involved in osmolyte synthesis, which contributes to ionic homeostasis.^45^ Potassium (K), meanwhile, is involved in the synthesis of various antioxidant compounds, including proline, phenolic compounds, and proteins. These compounds are associated with reduced membrane damage, as well as decreased levels of malondialdehyde (MDA) and H_2_O_2_. This fact is directly associated with potassium sulfate providing a reduction in free Na^+^ levels in cells through the activity of antioxidant enzymes, reducing oxidative damage and lipid peroxidation, and maintaining the stability of cell membranes.^46^

On the other hand, the increase in Na^+^ content observed with increasing salinity reduced foliar K^+^ levels since both ions compete for the same transport pathways. This reflects the antagonistic effect of excess Na^+^ in the irrigation water, resulting in ionic imbalance within plant cells.^47^ In contrast, the application of potassium sulfate resulted in a lower Na^+^/K^+^ ratio, enhancing the tolerance of cowpea to salt stress. This improvement can be attributed to better ionic homeostasis within the cells, facilitated by the potassium supply, which helped reduce Na^+^ accumulation and its associated phytotoxic effects.^48^ This ionic balance can occur thanks to the action of the presence of H_2_S, which regulates the expression of H^+^ATPase in the plasma membrane, enabling the maintenance of an H^+^ gradient that stimulates the efflux of Na^+^ and the influx of H^+^ to drive the Na^+^/H^+^ antiport across the plasma membrane, resulting in the extrusion and decreased absorption of Na^+^ ions.^49^

Nutritional management is an effective strategy for mitigating the effects of salt stress on agricultural crops, enabling cultivation with high electrical conductivity. Our study demonstrated that incorporating sulfates into fertilizer management represents a viable alternative for farmers in semiarid regions affected by salinity. Specifically, calcium and potassium sulfates improved both the antioxidant activity and overall performance of cowpea plants (cv. BRS Tumucumaque).

## 5 Conclusions

A salinity of 4.5 dS m^–1^ compromises the growth and productivity of cowpea plants (cv. BRS Tumucumaque). However, the application of calcium sulfate stimulated the synthesis of photosynthetic pigments. The application of sulfates, especially calcium and potassium sulfate, helped to alleviate the deleterious effects of saline stress, improving plant growth and productivity, increasing the activity of antioxidant enzymes, and reducing the negative impact of sodium on ionic balance
